# First-in-Human Segmental Esophageal Reconstruction Using a Bioengineered Mesenchymal Stromal Cell–Seeded Implant

**DOI:** 10.1016/j.jtocrr.2021.100216

**Published:** 2021-08-09

**Authors:** Johnathon M. Aho, Saverio La Francesca, Scott D. Olson, Fabio Triolo, Jeff Bouchard, Laura Mondano, Sumati Sundaram, Christina Roffidal, Charles S. Cox, Louis M. Wong Kee Song, Sameh M. Said, William Fodor, Dennis A. Wigle

**Affiliations:** a Division of General Thoracic Surgery, Department of Surgery, Mayo Clinic, Rochester, Minnesota; b Department of Physiology and Biomedical Engineering, Mayo Clinic, Rochester, Minnesota; c Biostage, Holliston, Massachusetts; d Department of Pediatric Surgery, McGovern Medical School, UTHealth, The University of Texas Health Science Center, Houston, Texas; e Gastroenterology and Hepatology, Mayo Clinic, Rochester, Minnesota; f Cardiovascular Surgery, Mayo Clinic, Rochester, Minnesota

**Keywords:** Esophageal reconstruction, Tissue engineered graft, Artificial organ, Esophageal replacement

## Abstract

**Introduction:**

Resection and reconstruction of the esophagus remains fraught with morbidity and mortality. Recently, data from a porcine reconstruction model revealed that segmental esophageal reconstruction using an autologous mesenchymal stromal cell-seeded polyurethane graft (Cellspan esophageal implant [CEI]) can facilitate esophageal regrowth and regeneration. To this end, a patient requiring a full circumferential esophageal segmental reconstruction after a complex multiorgan tumor resection was approved for an investigational treatment under the Food and Drug Administration Expanded Access Use (Investigational New Drug 17402).

**Methods:**

Autologous adipose-derived mesenchymal stromal cells (Ad-MSCs) were isolated from the Emergency Investigational New Drug patient approximately 4 weeks before surgery from an adipose tissue biopsy specimen. The Ad-MSCs were grown and expanded under current Good Manufacturing Practice manufacturing conditions. The cells were then seeded onto a polyurethane fiber mesh scaffold (Cellspan scaffold) and cultured in a custom bioreactor to manufacture the final CEI graft. The cell-seeded scaffold was then shipped to the surgical site for surgical implantation. After removal of a tumor mass and a full circumferential 4 cm segment of the esophagus that was invaded by the tumor, the CEI was implanted by suturing the tubular CEI graft to both ends of the remaining native esophagus using end-to-end anastomosis.

**Results:**

In this case report, we found that a clinical-grade, tissue-engineered esophageal graft can be used for segmental esophageal reconstruction in a human patient. This report reveals that the graft supports regeneration of the esophageal conduit. Histologic analysis of the tissue postmortem, 7.5 months after the implantation procedure, revealed complete luminal epithelialization and partial esophageal tissue regeneration.

**Conclusions:**

Autologous Ad-MSC seeded onto a tubular CEI tissue-engineered graft stimulates tissue regeneration following implantation after a full circumferential esophageal resection.

## Significance Statement

A biocompatible interposition graft facilitating esophageal regeneration would have considerable clinical use in short circumferential segment resection and reconstruction of the esophagus.

## Introduction

Several conditions that affect the esophagus may ultimately require surgical resection and reconstruction. These include cancer and precancerous lesions, congenital disorders such as esophageal atresia, functional disorders such as achalasia, refractory benign esophageal strictures, and nonhealing persistent esophageal fistulas or segmental necrosis.^1^, ^2^, ^3^, ^4^, ^5^ After esophageal resection, reconstructive options typically involve autologous conduits, such as the small bowel, colon, or a portion of the stomach (typically divided at the cardia to create a tubular structure), to reestablish foregut continuity. Despite improvements in surgical techniques and perioperative care, esophageal resection and reconstruction remains associated with significant morbidity and mortality.^1^, ^2^, ^3^, ^4^^,^^6^ Recently, alternative approaches to repair the esophagus using a tissue-engineered, in vitro-manufactured graft or commercially available stents that have been preconditioned to stimulate tissue growth have been described.^5^^,^^7^, ^8^, ^9^ Preclinical data in a large animal model of esophageal full-thickness circumferential defect replacement have revealed that this approach is a viable alternative for short-segment resection and reconstruction that stimulates the regeneration of a biological esophageal conduit.^7^^,^^8^ These studies used a tissue-engineered graft composed of polyurethane scaffolds that were seeded with three different cell types, epithelial cells,^7^ adipose-derived mesenchymal stromal cells (Ad-MSCs),^8^ or cells isolated from an esophageal biopsy specimen.^9^ Barron et al.^7^ described the regeneration of esophageal tissue; however, the contribution of the epithelial cells seeded onto the graft was unclear. La Francesca et al.^8^ described the use of autologous Ad-MSC–seeded grafts that stimulated full-thickness repair of the esophageal tissue and revealed full epithelialization of the luminal surface by 4 months postimplantation.

A single human case report describing the repair of a full circumferential esophageal defect with the use of a fully covered self-expanding metallic stent in combination with an AlloDerm patch (BioHorizons, formerly LifeCell), plus an autologous platelet-rich plasma and a sternocleidomastoid muscle flap, indicated that a segmental full circumferential esophageal defect could be repaired with restoration of oral nutritional intake.^5^ Nevertheless, the complex procedural steps and the extended use (>3 y) of three telescopically deployed overlapping stents are prohibitive to using such technique as a standard of care for patients in need of a segmental esophageal repair procedure.

In 2016, the Mayo Clinic (Rochester, MN) was presented with a 75-year-old male patient with a right lower lobe NSCLC. The patient was initially deemed inoperable on two previous surgical explorations secondary to mediastinal invasion into the heart and esophagus. Subsequent workup revealed no evidence of lymph node involvement or distant metastatic disease. Endoscopic ultrasound revealed that the distal esophagus was invaded covering a 3-cm segment. Esophageal reconstruction options were limited given previous small bowel and colon surgery, multiple laparotomies with abdominal mesh placement, and concern regarding the vascular supply necessary to support an intrathoracic autologous conduit. Given these issues, the patient was offered a right lung bilobectomy with atrial reconstruction and short-segment esophageal reconstructive option using an investigational combination product, the Cellspan esophageal implant (CEI), a tissue-engineered autologous Ad-MSC–seeded polyurethane graft (Biostage Inc., Holliston, MA). These surgical options were presented to the Mayo Clinic institutional review board (approval #17-002214) and the Food and Drug Administration (FDA), using the Expanded Access Use—Individual Patient Emergency Investigational New Drug (IND) application process (21 CFR 312.310). An expanded access use exemption IND was approved (IND #17402), and informed consent from the patient was obtained.

## Materials and Methods

### Adipose Cell Isolation

One month before the planned tumor removal operation, the patient underwent an abdominal adipose tissue biopsy for Ad-MSC isolation.

### Cellspan Esophageal Implant Manufacturing

The adipose tissue biopsy specimen was placed in a sterile container filled with α-minimal essential medium (α-MEM) supplemented with 50 μg/mL gentamycin, along with the patient’s blood samples for infectious disease marker testing, and shipped at 2°C to 8°C inside a temperature-controlled box, using a commercial airline, to UTHealth—The Judith R. Hoffberger Cellular Therapeutics Laboratory, a FDA— and Clinical Laboratory Improvement Amendments—registered, College of American Pathologists— and Foundation for the Accreditation of Cellular Therapy—accredited International Organization for Standardization 7 current Good Manufacturing Practice (GMP) facility in Houston, Texas. All procedures were compliant with FDA regulations and guidelines, and risk analysis of the manufacturing process was performed as previously described.^10^^,^^11^ In brief, the adipose tissue was rinsed in Ca++- and Mg++-free phosphate-buffered saline (PBS) supplemented with 50 μg/mL gentamycin until complete blood and blood clot removal and then weighed. Then, all connective tissues, lymph nodes, and blood vessels were trimmed off (final weight = 26.5 g). The sample was then minced into less than 5-mm pieces and digested for 30 minutes at 37°C in a 1:1 (v/v) ratio with current GMP-grade Liberase MNP-S (Roche Diagnostics GmbH, Penzberg, DE) at a final activity of 0.28 Wünsch U/mL. After stopping the digestion with MSC Expansion Media, α-MEM, supplemented with 20% fetal bovine serum Premium Select (Atlanta Biologicals, Flowery Branch, GA) and 1× GlutaMAX (Thermo Fisher Scientific, Grand Island, NY), the digested cell suspension was centrifuged at 400 *g* for 15 minutes at room temperature and the pellet was resuspended in the Expansion Media. The cell suspension was subsequently filtered through a 70-μm cell filter, recentrifuged as previously described, and resuspended in the Expansion Media in a volume equal to the weight of the initial biopsy (e.g., 30 g = 30 mL). The cell suspension was plated in 9 to 10 mL aliquots per T225 flask (passage 0) and incubated at 37°C in a humidified mixture of 95% air and 5% CO_2_. Nonadherent cells were removed after 2 to 3 days, and expansion medium was changed every 2 to 4 days thereafter. On reaching 70% to 90% confluence, the cells were rinsed with Ca++- and Mg++-free PBS, detached with TrypLE Select XenoFree reagent, transferred to Corning 5-STACK CellSTACKs at a density of 1000 to 2500 cells/cm^2^, and expanded to passage 3 (27 days population doubling level = 8.85) to reach the target number of cells (27.6 × 10^6^) needed to seed a 20 mm (internal diameter) × 110 mm (L) polyurethane scaffold (Biostage, Holliston, MA), at passage 3. The total cell yield at passage 3 from 8 CellSTACK parallel cell cultures equal to 1.24 × 10^8^ with a viability of 96%. Criteria for release of MSCs to seed the scaffold were as follows: greater than or equal to target cell number at passages 2 to 5, greater than or equal to 70% viability, plastic adherence and expected shape and size of human MSCs, negative Gram stain result, and less than 2 Endotoxin Units/mL. Characterization of the cells included the expression of CD34, CD45, CD31, HLA-DR, CD29, CD73, CD90, and CD105, which is consistent with conventional MSC phenotypes^10^, ^11^, ^12^, ^13^, ^14^ and confirmed the identity of the cells. In addition, the conditioned media was analyzed for the expression of proangiogenic molecules, vascular endothelial growth factor (VEGF), interleukin (IL)-8, and matrix metalloproteinase-2 (MMP2), which were used for potency criteria and to assign a mechanism of action for the combination product.

After homogeneously seeding the target cell number on the entire surface of the scaffold, the CEI was cultured for 6 days in expansion medium, rotating inside a bioreactor (Biostage, Holliston, MA), and incubated at 37°C in a humidified mixture of 95% air and 5% CO_2_. On days 2 and 4, aerobic and anaerobic sterility cultures were inoculated with conditioned medium, after which the medium was changed. On day 6, the scaffold was transferred from the bioreactor to a sterile transport chamber filled with expansion medium and airshipped overnight to the Mayo Clinic, at 2°C to 8°C inside a temperature-controlled box. Criteria for release of the scaffold for implantation were as follows: negative Gram stain result of day 6 conditioned medium, no microbial growth in aerobic and anaerobic cultures inoculated on days 2 and 4, less than or equal to 5 Endotoxin Units/kg of patient weight, no mycoplasma detection, and greater than or equal to 3 quadrants with live cells using the LIVE/DEAD Viability Cytotoxicity Kit (Thermo Fisher Scientific, Waltham, MA) on the basis of calcein and ethidium bromide staining. In brief, five 10-mm-diameter punches of a scaffold surrogate (an identical scaffold prepared under identical conditions, for quality control) were stained for viability on the basis of calcein green fluorescence with dead cells indicated by ethidium bromide (red) intercalation determined using digital analysis of fluorescence micrographs by quadrants.^7^^,^^8^

#### Metabolic Activity

Conditioned media samples taken at days 2, 4, and 6 harvest time points were analyzed for glucose and lactate content using an iSTAT portable blood analyzer (Abbott Diagnostics, Princeton, NJ). Metabolic readings were performed using G+ cartridges (measure glucose consumption) and CG4+ (measure lactate production and pH changes).

#### Flow Cytometry

Before use, the LSR II and Galios cytometers are powered on and allowed to initialize for a minimum of 30 minutes. Beckman Coulter Flow-Check beads (catalog #6605359) are used daily to standardize the instrument.

**Figure 1:**
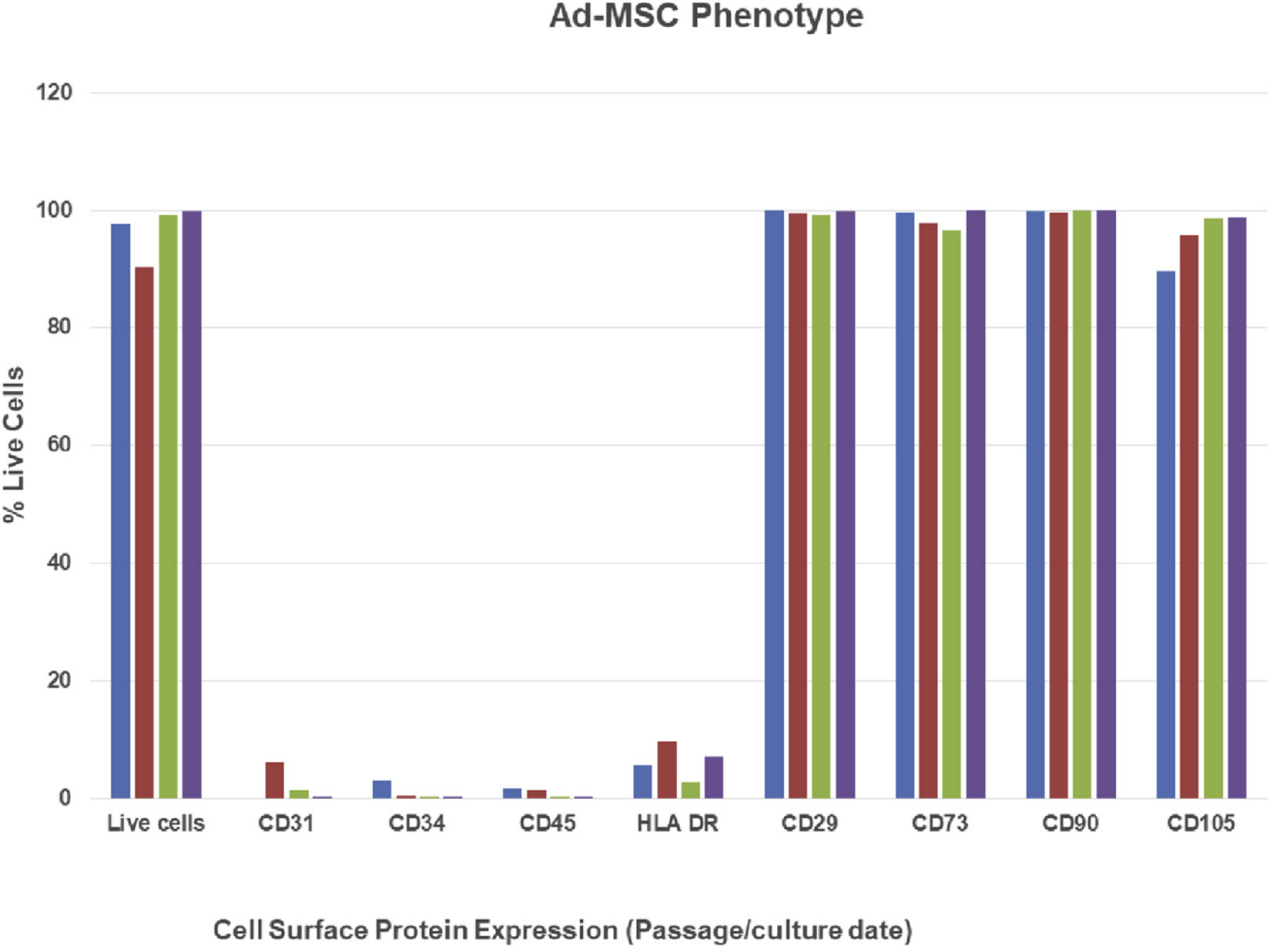
MSC cell surface phenotype. Flow cytometry of the Ad-MSCs was performed at the indicated passage time points during the manufacturing process. The percentage of live cells expressing the indicated cell surface marker is plotted for each passage. Ad-MSC, adipose-derived mesenchymal stromal cell; MSC, mesenchymal stromal cell.

The patient-specific autologous human MSCs were analyzed for expression of MSC surface markers^10^^,^^11^ and non-MSC lineage markers to characterize the cells before the production of the CEI (Fig. 1). The expanded Ad-MSCs were analyzed by flow cytometry at each passage for the expression of CD29, CD73, CD90, and CD105. The cells were also analyzed for the expression of CD31, CD34, CD45, and HLA-DR to ensure that there were no contaminating cell types before seeding the CEI (Fig. 1).

Adherent cells were washed twice in DPBS without Ca/Mg (HyClone) and dissociated with TrypLE Express (Gibco). The dissociated cells were quenched with growth medium and centrifuged at 400 *g* for 5 minutes. Cell pellet was resuspended in wash buffer and 1% bovine serum albumin (BSA Fraction V, 7.5%, Gibco) and diluted in DPBS (HyClone). A total of 1 million test cells and isotype controls were aliquoted and incubated in primary antibody at 4°C for 30 minutes in the dark. The labeled cells were washed three times in wash buffer, secondary antibodies were applied and incubated at 4°C for 30 minutes in the dark, and the cells were washed again three more times. The cells were aliquoted into the tubes and stained with antibodies against CD73, CD34, CD45, CD90, CD105, and HLA-DR with 7-aminoactinomycin D to compose one panel and CD29, CD31 to compose a second panel, all following the manufacturer’s instructions at a dilution of 1:100. Viability was determined with the addition of 7-aminoactinomycin D. After a 30-minute incubation with antibodies, the cells were washed once with PBS and a second time with a staining buffer before final resuspension in 0.3 mL of a staining buffer. The samples were prepared along with unstained control cells. Data were acquired from a minimum of 10,000 events collected in a single acquisition. Data were exported and analyzed by standalone software (FlowJo version 10, FlowJo, LLC, Ashland, OR).

#### Enzyme-Linked Immunosorbent Assay Analysis

**Figure 2:**
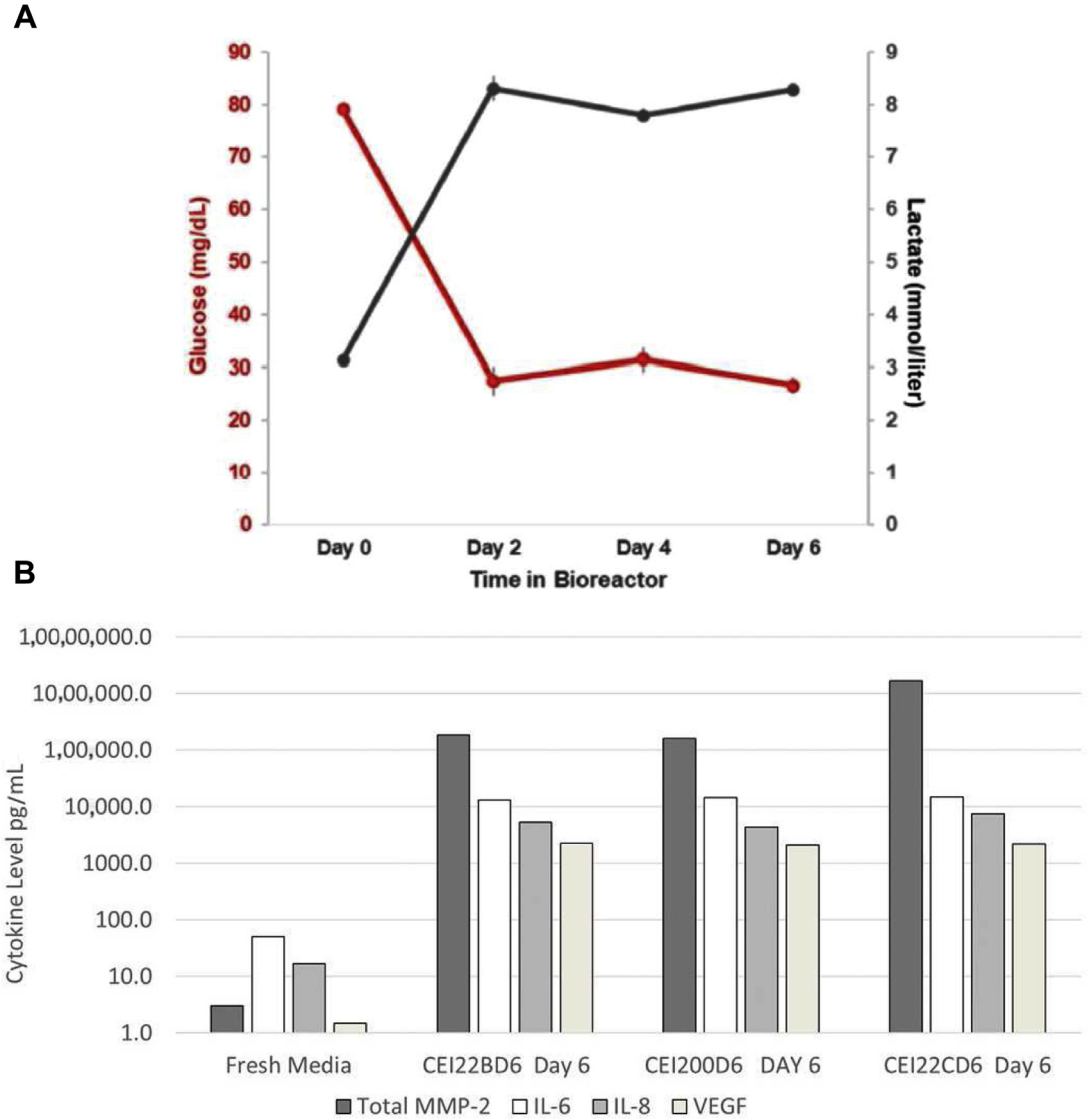
Conditioned media analysis, glucose/lactate MSC cytokine analysis. (*A*) Conditioned media was analyzed for glucose and lactate levels using an iSTAT. (*B*) ELISA analyses determined the cytokine/growth factor concentrations in the conditioned media at the time of CEI harvest. CEI, Cellspan esophageal implant; ELISA, enzyme-linked immunosorbent assay; IL, interleukin; MMP, matrix metalloproteinase; MSC, mesenchymal stromal cell; VEGF, vascular endothelial growth factor.

Day 6 conditioned media was removed from the bioreactors during scheduled media change and analyzed for VEGF, the cytokines, IL-6 and IL-8, and MMP2 secretion. Enzyme-linked immunosorbent assay (ELISA) assays were performed at the University of Minnesota as described (Fig. 2^8^).

### Results: Postoperative Course

After the isolation of the autologous Ad-MSC from the biopsy tissue (trimmed weight = 26.5 g), the cells were cultured in 2-dimensional (2D) tissue culture flasks and CellSTACK culture vessels using a GMP-grade culture media (Expansion Media; α-MEM + 20% fetal bovine serum + 1% GlutaMax). Characterization of the Ad-MSC follows the standards,^10^, ^11^, ^12^ in which Ad-MSCs are characterized by plastic adherence, exponential proliferation under MSC growth conditions, cell surface epitopes using flow cytometry, immunomodulatory activity in activated leukocyte co-cultures, and trophic cytokine expression and secretion.^15^

Cells were characterized by flow cytometry at each passage harvest to ensure that the cells were expressing CD73, CD90, and CD105, known MSC cell surface proteins (Fig. 1). In addition, the cells were negative for the endothelial cell marker, CD31, the HSC marker CD34, human leukocyte antigen CD45, and histocompatibility antigen HLA-DR (Fig. 1). The cell phenotype was consistent throughout the 2D culture period where the expressions of CD73, CD90, and CD105 were greater than 90% at each passage harvest (Fig. 1). The overall growth potential of the Ad-MSCs harvested from 26.5 g of adipose tissue (75-y-old male) based on the P0 harvest of 4.5 × 10^6^ cells cultured to passage 3 (population doubling level = 8.38; Supplementary Table 3 and Supplementary Fig. 1B) was approximately 1.1 × 10^9^ total cells.

After 2D cell culture expansion, the cells were seeded onto the CEI scaffold at a density of 4000 cells/mm^2^ and cultured in a custom bioreactor for 6 days. During the 6-day incubation period, media was exchanged at days 2 and 4 postseeding and assayed for glucose utilization and lactate production to evaluate metabolic activity and viability (Fig. 2*A*). The conditioned media from the bioreactor was also tested to determine the level of the angiogenic protein factors secreted from the Ad-MSCs, including VEGF, IL-8, and MMP2. ELISA analysis of the conditioned media determined that all three factors were secreted from the CEI (Fig. 2*B*). The final CEI product was harvested at the 6-day time point postbioreactor incubation and transferred to a dedicated shipping tube for shipment to the surgical facility. Sterility and endotoxin analysis (purity) were performed, and data were reviewed retrospectively. All test data on the CEI met the release criteria, including sterility and purity.

#### Operative Intervention

**Figure 3:**
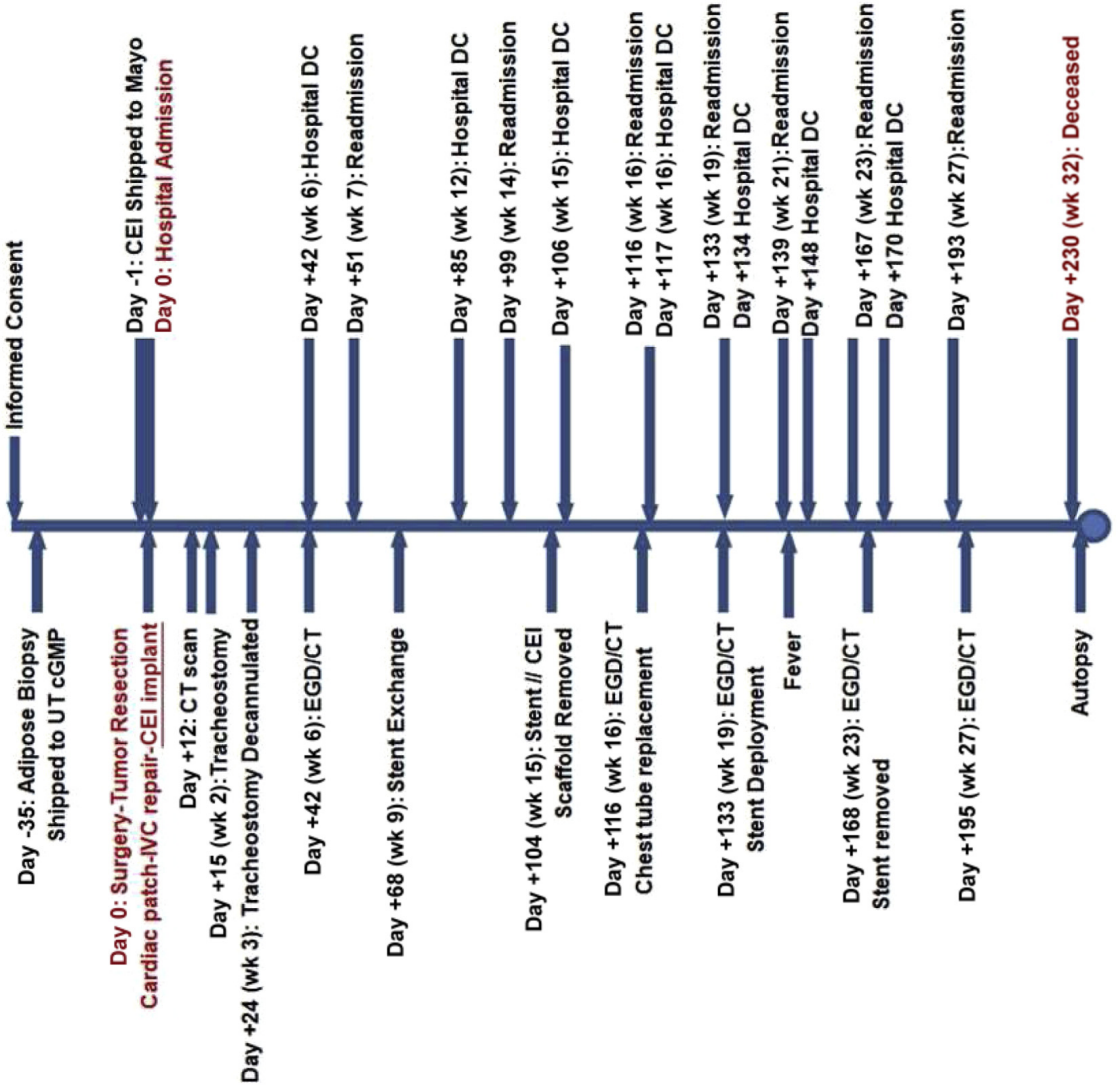
Patient time course illustrated in a linear timeline with procedural interventions and hospital admission/DC times. CEI, Cellspan esophageal implant; cGMP, current Good Manufacturing Practice; CT, computed tomography; DC, discharge; EGD, esophagogastroduodenoscopy; UT, The University of Texas.

**Figure 4:**
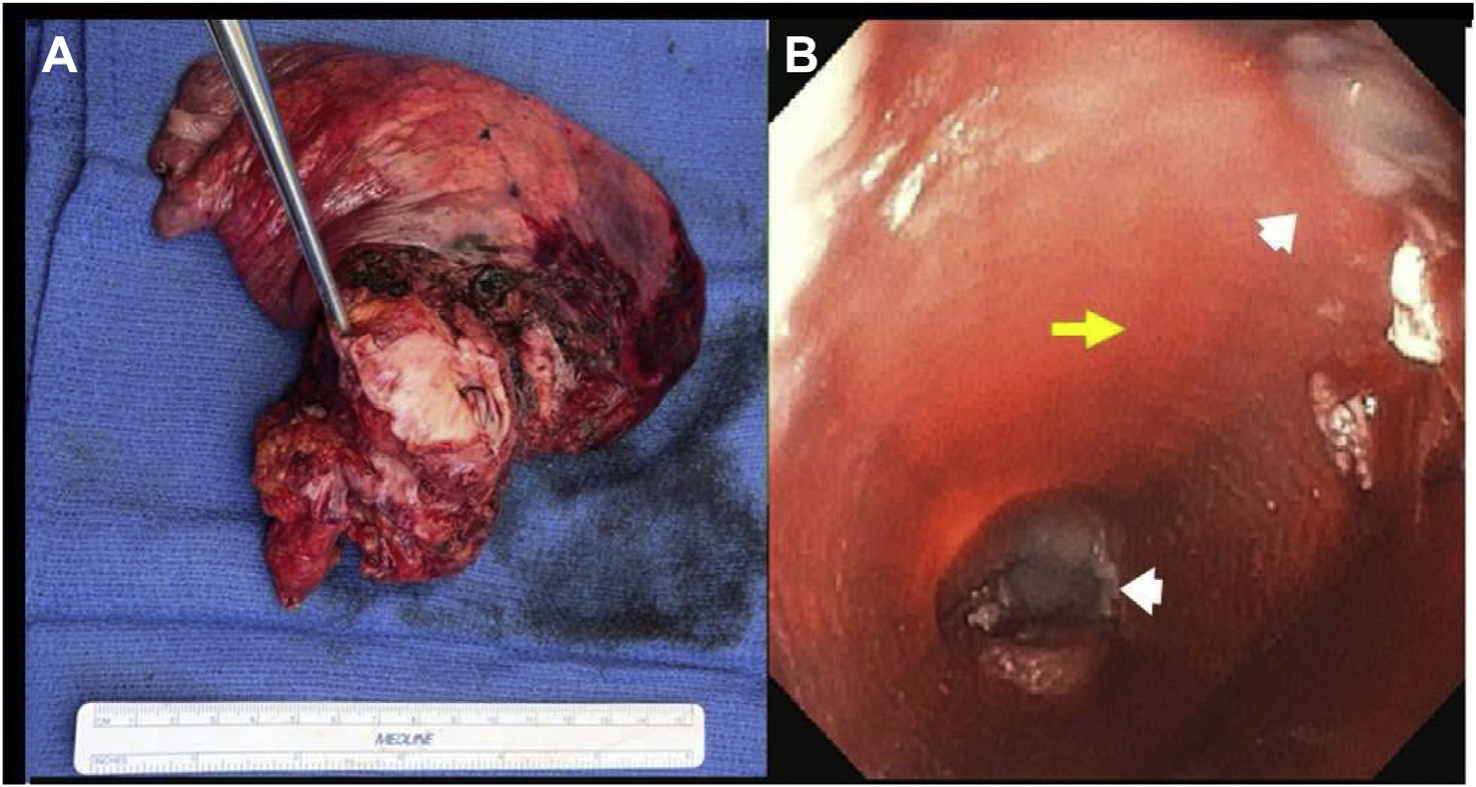
(*A*) Gross surgical specimen of the tumor removed, including the lung, portion of the inferior vena cava, both atria, and the esophagus. (*B*) Endoscopic view before stent deployment of the esophageal implant (yellow arrow). The proximal and distal suture lines between the graft and the native esophagus are visible (white arrowheads).

The patient time course is illustrated in Figure 3. After Emergency IND approval from the FDA and subsequent enrollment, the patient underwent an abdominal adipose tissue resection to isolate tissue for Ad-MSC isolation and expansion 5 weeks before the implant procedure of the CEI (d −56). On day −1 before the tumor resection and implant surgery of the CEI, the patient underwent an esophagogastroduodenoscopy (EGD) with gastrostomy tube placement for postoperative enteral feeding and venting. Computed tomography (CT) scan of the chest without intravenous contrast indicated no significant change in the right lower lobe mass. There was no significant change in size or appearance of the soft tissue mass in the right lower lobe medially which measured 5.3 × 3.1 cm. The mass was contiguous and invasive into the distal esophagus, left atrium, and right inferior pulmonary vein with loss of fat planes between it and adjacent structures. The patient then underwent a thoracotomy procedure to remove the tumor using a right hemi clamshell incision with resection of right lower and middle lung lobes. A portion of the left and right atriums of the heart, a portion of the inferior vena cava (IVC), and the involved esophagus also required resection (4 cm in length circumferentially; Fig. 4*A*). Gross examination of the excised tumor tissue revealed multiorgan extent of invasion by the tumor (Fig. 4*A*). Esophageal reconstruction was performed during cardiopulmonary bypass, using the CEI esophageal graft implant trimmed to match the resection (∼5 cm) (Fig. 4*B*). Reconstruction of the left and right atriums was performed using a bovine pericardial patch. The IVC was reconstructed with a GORE-TEX Vascular Graft (W.L. Gore & Associates, Inc., Flagstaff, AZ). A pedicled pericardial fat pad was interposed between the esophageal reconstruction and the other areas of reconstruction, and a fully covered esophageal stent (Boston Scientific, Marlborough, MA) was placed at the end of the entire procedure. Total cardiopulmonary bypass time was 267 minutes, with an aortic cross clamp time of 92 minutes. Postsurgical CT scan result revealed no evidence of the tumor in the chest and no evidence of lymphadenopathy.

#### Postoperative Course

**Figure 5:**
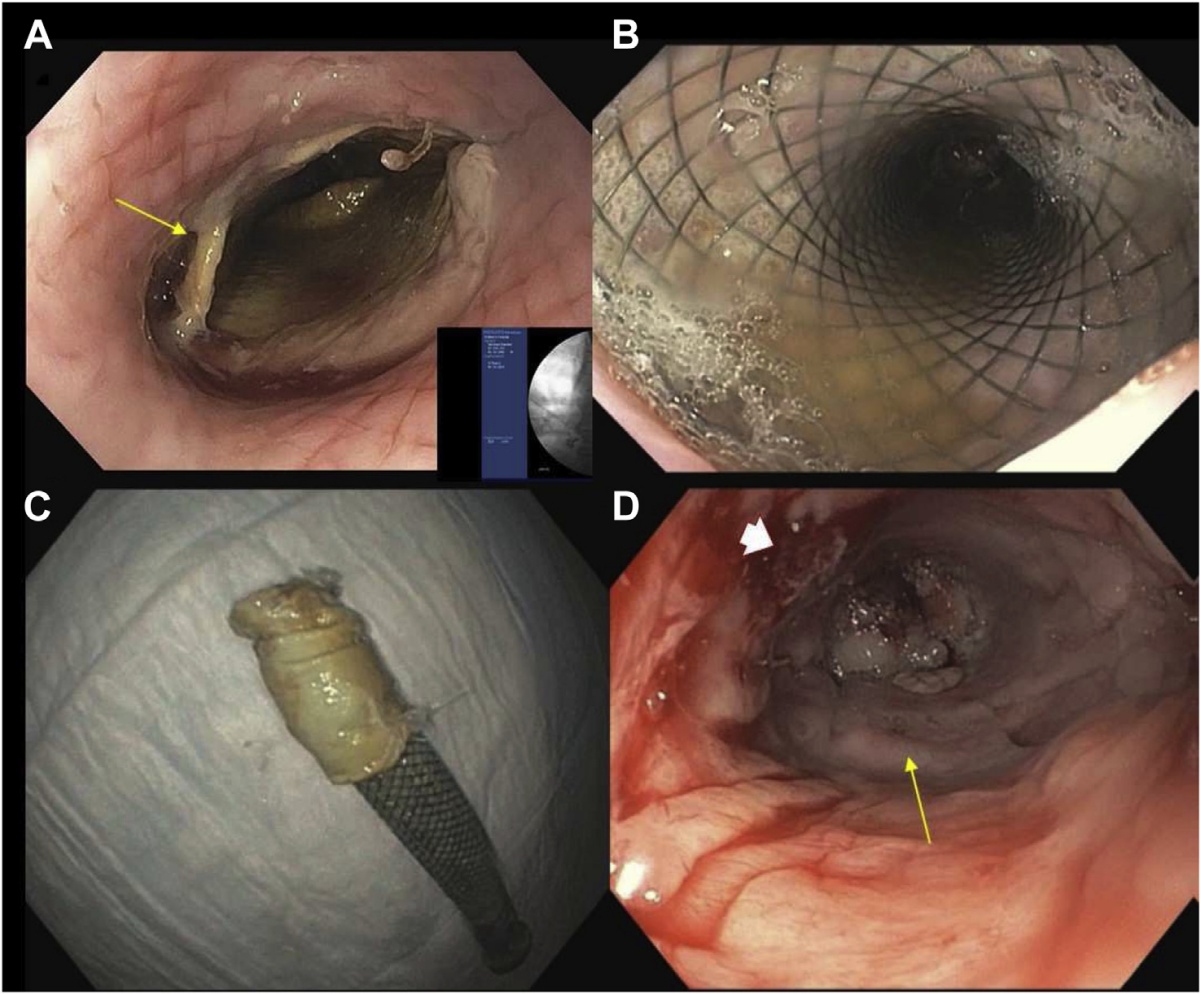
Endoscopic imaging of the esophagus and scaffold/stent removal. (*A*) EGD image of the esophagus after stent removal (d +68) and proximal aspect of the CEI where the scaffold is beginning to separate from the tissue (yellow arrow). (*B*) EGD image of the esophagus after stent redeployment (d +68). (*C*) Removed second stent with the removed CEI scaffold component adhering to the stent (d +104). (*D*) EGD image of the epithelialized CEI implant zone neotissue at day +104 postsurgery (yellow arrow). Pleural fistula indicated by white arrowhead. CEI, Cellspan esophageal implant; EGD, esophagogastroduodenoscopy.

The patient recovered as anticipated in the early postoperative period. There was no evidence of reaction to the graft or other immunoinflammatory response beyond that expected from a major operation. The patient was initially on vasoactive medication for hemodynamic support and was subsequently weaned. Enteric tube feeding was initiated and titrated to caloric goal. The patient was extubated to bilevel positive airway pressure, and he developed atrial fibrillation with rapid ventricular response that required pharmacologic treatment. After 12 days from surgery, a follow-up CT examination without intravenous contrast indicated interval extensive changes in the chest related to resection of the lung carcinoma in the medial right lower lobe and invading the right hilum and mediastinum. A moderate-sized right pleural effusion was also observed. A small amount of patchy cellular infiltrate, primarily in the right upper lobe, was assumed to be infectious/inflammatory in nature. In addition, a small area of pleural effusion and atelectasis was observed in the left lung base. Regarding the CEI implant region, there was no evidence of fluid along the course of the anterior mediastinal drain and the distal esophageal stent seemed widely patent. The patient developed acute respiratory failure after extubation, requiring subsequent tracheotomy (postoperative d +15) for ventilatory management and secretion clearance. The patient was weaned off mechanical support on day +24. Six weeks after the surgery (d +42), the patient underwent upper endoscopy and CT scans to evaluate the implant. The esophageal stent was well positioned within the implant. The endoscopy revealed areas of tissue regrowth with normal mucosa where the implant graft started to naturally separate from the esophageal wall. The patient was then discharged from the hospital (day +42). CT scan results and evaluation concluded the existence of a moderate right loculated hydropneumothorax with the presence of gas tracking along the posterior mediastinum to the esophageal stent, potentially indicating the development of an esophageal-pleural fistula. The patient was readmitted nine days after the initial discharge (d +51) and underwent follow-up CT and EGD examinations. Similar to the previous findings, a moderate, loculated right hydropneumothorax, extending into the posterior mediastinum was observed. The air tracks along the right aspect of the esophageal stent indicated the presence of a fistula or an esophageal leak from the proximal end of the stent near the proximal suture line. These findings prompted the decision to replace the esophageal stent without disrupting or removing the CEI scaffold by means of EGD (d +68) (Fig. 5*A* and *B*). The patient was then discharged on day +85.

Approximately 3 months from the surgery, the patient was readmitted (d +99) for symptoms associated with an esophageal leak (turbid chest tube discharge). A follow-up EGD was performed (d +104) to remove the esophageal stent and the polyurethane CEI scaffold device component. Similar to the observations from several large animal studies,^8^ the esophageal scaffold component of the CEI graft naturally separated from the newly grown esophageal conduit and was easily retrieved along with the esophageal stent (Fig. 5*C*). The OverStitch sutures that anchored the stent were sectioned using argon plasma coagulation. Stent removal with the adhered polyurethane scaffold was performed with a large, rat-toothed forceps. Regenerated neoesophageal tissue was noted in the implant region of the lower esophagus and seemed to be fully epithelialized, as evident by a luminal glossy mucosal lining (Fig. 5*D*) that was grossly viable in appearance (Fig. 5*D*). In addition a 1-cm fistula tract to the right side of the chest that was delineated after contrast injection was noted. The fistula was filled with fibrin glue (10 mL) (Baxter, Deerfield, IL), and the patient was discharged on the next day without deploying a replacement stent owing to patient discomfort and intermittent chest pain with the stent in place.

The patient was readmitted for chest tube replacement and follow-up CT scan at approximately 4 months postsurgery (d +116). The CT scan revealed a patulous esophagus with circumferential mural thickening in the mid and distal segments. An air-filled tract from the esophageal lumen at the level of the left atrium was in continuity with a more organized moderate-sized complex right pleural exudate, which was predominantly air filled with trace-dependent fluid consistent with the esophageal-pleural fistula tract. The patient was discharged on the next day. Diet was maintained through enteric feeding tube and small amounts orally for comfort only.

Approximately 4.5 months postsurgery (d +133), the patient was again readmitted for symptoms related to the esophageal leak (infection or fever). An EGD was performed to evaluate the CEI and esophageal-pleural fistula. The EGD revealed a stricture in the mid-distal esophagus measuring approximately 6 mm in diameter. Adjacent to the stricture, the persistent fistula could be identified and seemed smaller in size (0.5 cm). The distal esophagus was examined with a pediatric endoscope and seemed unremarkable (epithelialized, white glossy mucosal appearance) within the CEI implant region. A stent was redeployed across the stricture and fistula. The OverStitch suturing system was used to anchor the proximal flange of the stent to the esophageal wall. Contrast fluoroscopy after stent deployment revealed freely flowing contrast agent through the stent into the stomach without apparent leak at the known fistula site. The patient was discharged on the next day (d +134).

The patient was readmitted for follow-up EGD and CT scan procedures at 5.5 months (d +167) postoperatively. The upper endoscopic procedures revealed gradual improvement in the fistula and maintenance of epithelization of the new esophageal tissue. Consistent with the EGD observation, the CT scan revealed a decreased size of the complex right posterior pleural collection from the retrograde pigtail catheter that traversed the known right esophageal-pleural fistula. In addition, persistent right lower lung atelectasis and mild left basilar atelectasis were observed with mild mediastinal edema. The patient was discharged 3 days after (d +170).

The patient was readmitted approximately 6.5 months postsurgery (d +193) after experiencing seizure activity. A confirmatory magnetic resonance imaging verified acute cerebrovascular attacks with potential superimposed cerebral infection, prompting the initiation of broad-spectrum intravenous antibiotics. A CT scan result of the thoracic region indicated no significant change in the complex right pleural collection with stable positioning of the retrograde transgastroesophageal-internalized pleural pigtail drainage catheter, which was effectively controlling the esophageal-pleural bronchial fistula. The patient continued to be febrile throughout this hospitalization period. Despite aggressive antimicrobial therapy, the patient continued to be febrile. Result from a magnetic resonance imaging of the head was suggestive of central nervous system vascular infection and leptomeningeal enhancement. Because of the profound, persistent neurologic deficits after acute ischemic stroke, the family ultimately decided to transition to comfort care. Antimicrobials were discontinued on day +228 at the time of initiation of comfort care. The patient expired approximately 7.5 months (d +230) after his operation for tumor resection, IVC and cardiac repair, and esophageal reconstruction using the CEI tissue-engineered graft. Autopsy result revealed the cause of death to be owing to multiple acute ischemic strokes, cerebral hemorrhage of the left frontal lobe, and multiple microemboli involving the brainstem, thalamus, amygdala, and occipital lobe, bilaterally, with likely superimposed bacteremia and central nervous system infection.

#### Gross Examination and Histopathology of the Esophagus

**Figure 6:**
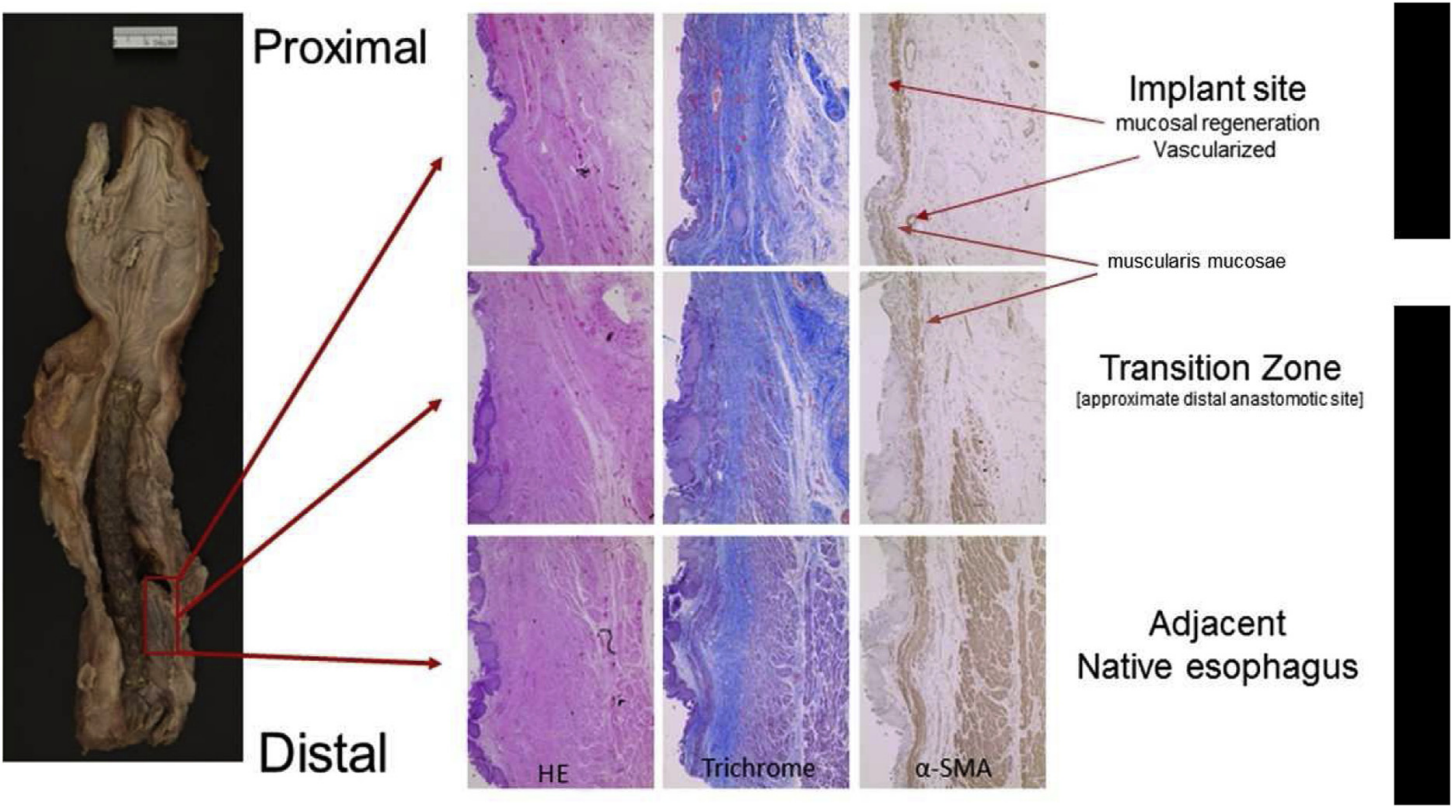
Image of the esophagus reveals regrowth of the esophageal mucosal layer and soft tissue structure along the length of the esophagus implant with the autologous adipose-derived stem cell-seeded graft (red box). Throughout the esophageal length, the gross structure and morphology of the esophagus were regrown. Individual panels revealing HE-, trichrome-, and α-SMA–stained sections. α-SMA, α-smooth muscle actin; HE, hematoxylin and eosin.

**Figure 7:**
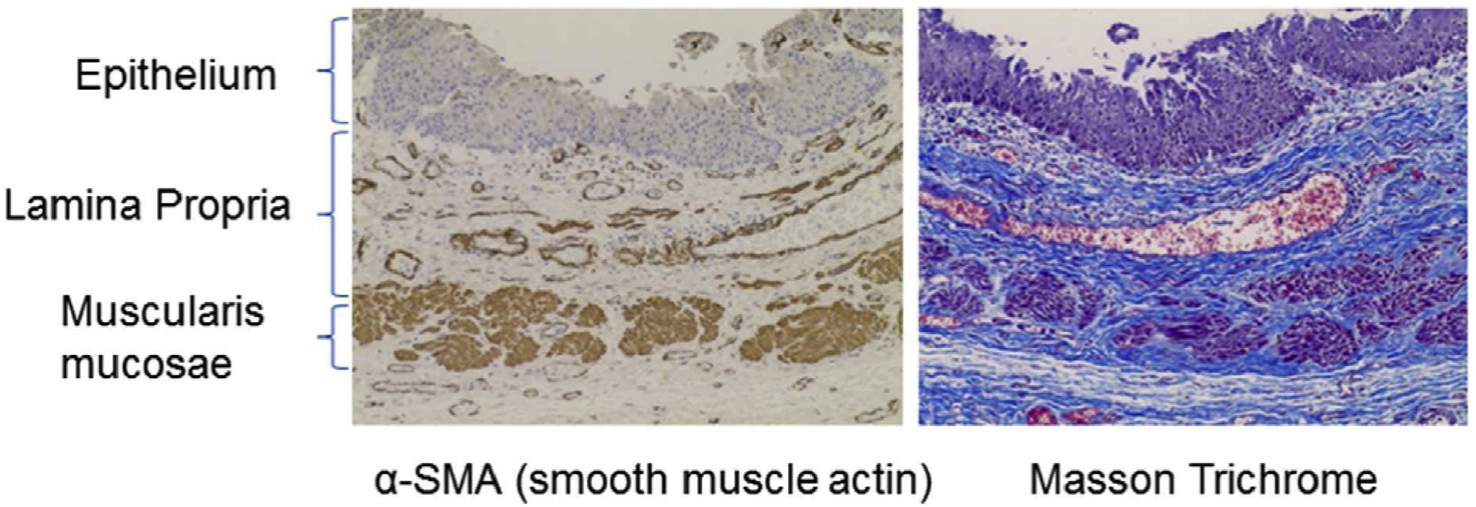
Mucosal regeneration with histologically similar morphology to normal thoracic esophageal tissue was observed in the transition zone from esophageal tissue specimens taken from the CEI implant zone, including glandular structures and smooth muscle. α-SMA, α-smooth muscle actin; CEI, Cellspan esophageal implant.

Gross examination of the explanted esophagus with the stent in place is illustrated in Figure 6. Histologic evaluation of different regions of the esophagus at the CEI implant site revealed the regions corresponding to native tissue and regions corresponding to the implant zone (Fig. 6). The native esophagus, the transition zone, and the center of the implant regions are clearly identifiable in the hematoxylin and eosin and trichrome sections on the basis of the level of fibrosis and the presence or absence of distinct tissue layers. The region identified as the “transition zone” reveals the regeneration of the epithelial layer on the luminal surface, regeneration of the muscularis mucosae, and initial regeneration of the tunica muscularis (α-smooth muscle actin panel; Fig. 7) with apparent muscularis migration toward the center of the implant region. The central portion of the regenerated tissue is clearly devoid of a complete tunica muscularis; however, the epithelial layer is complete with an associated complete muscularis mucosae (α-smooth muscle actin panel; Fig. 7).

## Discussion

To our knowledge, this case represents the first-in-human application of a clinical-grade, tissue-engineered esophageal graft composed of a synthetic polyurethane scaffold and seeded with autologous Ad-MSCs. This case reveals that such an approach has potential to be used clinically for segmental full-circumferential esophageal reconstruction. Results in this single patient suggest that the CEI manufacturing process steps, including cell isolation, expansion, and seeding, of the Ad-MSCs on the graft are achieved within the necessary time frame for actual clinical use. Interestingly, the growth potential (Supplementary Fig. 1*A*) for the harvested Ad-MSC from a 75-year-old patient produced sufficient numbers of cells with a consistent phenotype (Fig. 2*A*) to seed multiple scaffolds (potential to seed >35 scaffolds). This case also reveals that the cell-seeded CEI, along with a back-up scaffold, can be shipped from the manufacturer to the hospital for the implant, in a timely and safe manner.

The patient was a complicated surgical case and clearly had a complicated postoperative course (Fig. 3) and ultimately expired; however, a fundamental finding of this study is that the cell-seeded scaffold was able to trigger enough esophageal tissue regeneration to reconstitute the integrity and continuity of the esophageal tube after a full-circumferential segmental resection. We believe that in this particular clinical setting of malignant invasion of the esophagus, clear resection to negative gross margins was required to provide complete resection as opposed to a lesser resection that may have allowed primary esophageal reconstruction. Furthermore, the regeneration process did occur in a manner consistent with the previous results obtained in a large animal model^7^^,^^8^ (Figs. 6 and 7). This study, in combination with previously published results of esophageal regeneration using the CEI tissue-engineered graft (S. Sundaram, unpublished data, 2020),^8^^,^^9^ confirms that the regeneration process is reproducible in humans. In addition, the data presented here confirm that epithelial regeneration occurs during the initial wave of tissue regeneration and is typically complete by 3 months postimplantation. Another common result observed between the large animal studies (S. Sundaram, unpublished data, 2020)^7^^,^^8^ is the neovascularization of the graft, suggesting angiogenesis plays a critical role in stimulating the regeneration/wound healing response after CEI implantation. To that end, characterizing the secretome of the Ad-MSCs is an important criterion that can be used to evaluate potency of the CEI before implantation (i.e., defining the angiogenic potential). Further studies, aimed at evaluating all mechanistic steps of esophageal regeneration, similar to studies reviewed by Fu et al.,^15^ will be critical to understanding the full potential of applying this technology as a treatment option for patients requiring esophageal reconstruction. Furthermore, as more of these approaches to reconstruction are performed, opportunities exist for refinement in standardization of postoperative care.

In conclusion, we have revealed the repair of an esophageal large-circumferential defect using a synthetic polymer-based scaffold seeded with autologous mesenchymal stem cells. Further clinical application of this technology to short-segment esophageal reconstruction seems feasible.

## CRediT Authorship Contribution Statement

**Saverio La Francesca:** Literature search, Study design, Data collection, Data analysis, Data interpretation, Writing, Critical revision.

**Johnathon M. Aho:** Literature search, Data analysis, Data interpretation, Writing, Critical revision.

**Sumati Sundaram:** Data collection, Data analysis, Data interpretation.

**Dennis A. Wigle:** Study design, Writing, Critical revision.

## References

[1] Kaman L., Iqbala J., Kundilb B., Kochharc R. (2010). Management of esophageal perforation in adults. Gastroenterology Res.

[2] van Boekel P.G., Siersema P.D. (2015). Refractory esophageal strictures: what to do when dilation fails. Curr Treat Options Gastroenterol.

[3] Dias E., Santos-Antunes J., Macedo G. (2019). Diagnosis and management of acute esophageal necrosis. Ann Gastroenterol.

[4] Dhir R., Sutcliffe R.P., Rohatgi A., Forshaw M.J., Strauss D.C., Mason R.C. (2008). Surgical management of late complications after colonic interposition for esophageal atresia. Ann Thorac Surg.

[5] Dua K.S., Hogan W.J., Aadam A.A., Gasparri M. (2016). In-vivo oesophageal regeneration in a human being by use of a non-biological scaffold and extracellular matrix. Lancet.

[6] Flanagan J.C., Batz R., Saboo S.S. (2016). Esophagectomy and gastric pull-through procedures: surgical techniques, imaging features, and potential complications. Radiographics.

[7] Barron M.R., Blanco E.W., Aho J.M. (2018). Full-thickness oesophageal regeneration in pig using a polyurethane mucosal cell seeded graft. J Tissue Eng Regen Med.

[8] La Francesca S., Aho J.M., Barron M.R. (2018). Long-term regeneration and remodeling of the pig esophagus after circumferential resection using a retrievable synthetic scaffold carrying autologous cells. Sci Rep.

[9] Jensen T., Wanczyk H., Sharma I., Mitchell A., Sayej W.N., Finck C. (2019). Polyurethane scaffolds seeded with autologous cells can regenerate long esophageal gaps: an esophageal atresia treatment model. J Pediatr Surg.

[10] Lopez F., Bartolo C. D.i., Piazza T. (2010). A quality risk management model approach for cell therapy manufacturing. Risk Anal.

[11] Katz N., Kukharenko V., Marvin J. Optimization of adipose-derived mesenchymal stem cells harvest protocol toward clinical applications. Roche Diagnostics Corporation. https://www.researchgate.net/profile/Nathan-Katz-3/publication/282662615_Optimization_of_Adipose-_Derived_Mesenchymal_Stem_Cells_Harvest_Protocol_Toward_Clinical_Applications/links/5616e07708ae90469c611b59/Optimization-of-Adipose-Derived-Mesenchymal-Stem-Cells-Harvest-Protocol-Toward-Clinical-Applications.pdf.

[12] Horwitz E.M., Le Blanc K., Dominici M. (2005). Clarification of the nomenclature for MSC: the International Society for Cellular Therapy position statement. Cytotherapy.

[13] Dominici M., Le Blanc K., Mueller I. (2006). Minimal criteria for defining multipotent mesenchymal stromal cells. The International Society for Cellular Therapy position statement. Cytotherapy.

[14] Mushahary D., Spittler A., Kasper C., Weber V., Charwat V. (2018). Isolation, cultivation, and characterization of human mesenchymal stem cells. Cytometry A.

[15] Fu Y., Karbaat L., Wu L., Leijten J., Both S.K., Karperien M. (2017). Trophic effects of mesenchymal stem cells in tissue regeneration. Tissue Eng Part B Rev.

