# Supplementary material for: First-in-Human Segmental Esophageal Reconstruction Using a Bioengineered Mesenchymal Stromal Cell–Seeded Implant
Source: JTO Clin Res Rep. 2021 Aug 9;2(9):100216. doi: 10.1016/j.jtocrr.2021.100216 (PMC8474397; doi:10.1016/j.jtocrr.2021.100216)
Supplement: Supplemental Figure 1 and Tables 1-3 [file mmc1.docx]

**Supplemental Tables and Figures**

Table S1. Antibody clones used for FLOW Cytometry

| Antibody-Fluor | Manufacturer | Clone |
| --- | --- | --- |
| 7-AAD | Invitrogen | - |
| Anti-human CD34 APC | BD Biosciences | 581 |
| Anti-human CD45 APC Cy7 | Biolegends | HI30 |
| Anti-human CD90 BV421 | BD Biosciences | 5E10 |
| Anti-human HLA DR BV510 | Biolegends | L243 |
| Anti-human CD73 PE | BD Biosciences | AD2 |
| Anti-human CD105 FITC | BD Biosciences | 266 |
| Anti-human CD29 PE | Invitrogen | TS2/16 |
| Anti-human CD31 APC | BD Biosciences | M89D3 |

Table S2. Cell surface protein expression at each cell culture passage

| **Passage** | **Date** | **Live cells** | **CD31** | **CD34** | **CD45** | **HLA DR** | **CD29** | **CD73** | **CD90** | **CD105** |
| --- | --- | --- | --- | --- | --- | --- | --- | --- | --- | --- |
| 0 | 4/7/17 | 97.72 | N/A | 3.1 | 1.69 | 5.63 | 99.98 | 99.58 | 99.73 | 89.63 |
| 1 | 4/14/17 | 90.33 | 6.11 | 0.4 | 1.44 | 9.86 | 99.44 | 97.91 | 99.55 | 95.89 |
| 2 | 4/20/17 | 99.16 | 1.46 | 0.04 | 0.09 | 2.91 | 99.23 | 96.67 | 99.96 | 98.63 |
| 3 | 4/27/17 | 99.78 | 0.26 | 0.03 | 0.03 | 7.13 | 99.89 | 99.93 | 99.99 | 98.81 |

Table S3. Cell population growth characteristics

|  | PDs | PDL | Doubling Time | Cells seeded | Cells harvested | Fold Expansion | Total Cells (projected) | Days in culture | Days post isolation |
| --- | --- | --- | --- | --- | --- | --- | --- | --- | --- |
| p0 |  | 1 |  |  | 4500000 |  | 4500000 | 8 | 8 |
| p1 | 3.1 | 4.1 | 54.78 | 3180000 | 26650000 | 8.3805 | 37712264.2 | 7 | 15 |
| p2 | 2.7 | 6.8 | 53.21 | 6360000 | 41500000 | 6.52516 | 246078453 | 6 | 21 |
| p3 | 1.58 | 8.38 | 78.12 | 28000000 | 124320000 | 4.44 | 1092588332 | 7 | 28 |

Figure S1. Cell culture characteristics


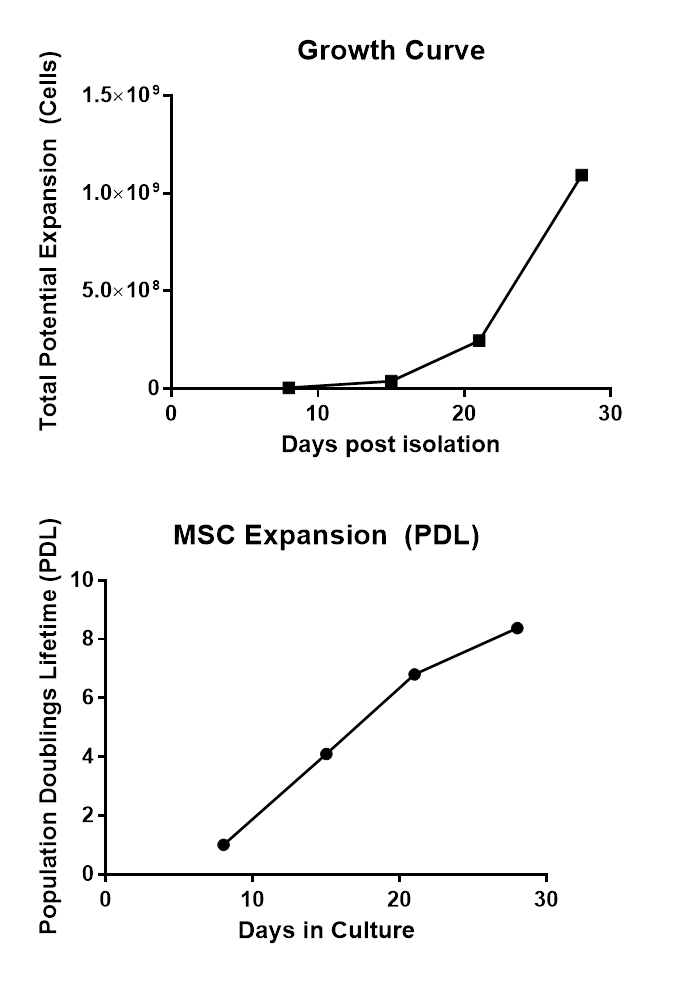


Population Doubling Level (PDL)

A

B
